# Intravenous branched-chain amino acid administration for the acute treatment of hepatic encephalopathy: a systematic review and meta-analysis

**DOI:** 10.1186/s40560-024-00771-x

**Published:** 2025-01-09

**Authors:** Shoji Yokobori, Tomoaki Yatabe, Yutaka Kondo, Yasuhiko Ajimi, Manabu Araki, Norio Chihara, Masao Nagayama, Tetsuya Samkamoto, the Japan Resuscitation Council (JRC) Neuroresuscitation Task Force and the Guidelines Editorial Committee, Hitoshi Kobata, Hajime Yoshimura, Michi Kawamoto, Masahiro Wakasugi, Hiroshi Yamagami, Hidetoshi Nakamoto, Eisei Hoshiyama, Kenichi Todo, Masaya Togawa, Mana Kurihara, Takashi Moriya, Ryuta Nakae, Hidetoshi Uchida, Sunghoon Yang, Masaaki Iwase

**Affiliations:** 1 Department of Emergency and Critical Care Medicine, Nippon Medical School, Bunkyo-Ku, Tokyo 113-8603 Japan; 2 Emergency Department, Nishichita General Hospital, Tokai-Shi, Aichi 477-8522 Japan; 3 Department of Emergency and Disaster Medicine, Juntendo University Graduate School of Medicine, Bunkyo-Ku, Tokyo 113-8431 Japan; 4 Department of Emergency Medicine, Teikyo University School of Medicine, Itabashi-Ku, Tokyo 173-8605 Japan; 5 Department of Neurology, Kawakita General Hospital, Tokyo, 166-8588 Japan; 6 Division of Neurology, Kobe University Hospital, 7-5-2, Kusunoki-Cho, Chuo-Ku, Kobe, 650-0017 Japan; 7 Departments of Neurology, Critical Care Medicine, and the Center for Preventive Medicine, International University of Health and Welfare Narita Hospital, Narita, Chiba 286-8520 Japan; 8 Showa General Hospital, Kodaira-Shi, Tokyo 187-8510 Japan

**Keywords:** Hepatic encephalopathy, Branched-chain amino acid, Acute care, Randomized controlled trial, Meta-analysis, Systematic review, Intravenous infusion

## Abstract

**Background:**

Hepatic encephalopathy (HE) is a severe complication of acute hepatic failure requiring urgent critical care management. Branched-chain amino acids (BCAAs) such as leucine, isoleucine, and valine have been investigated as potential treatments to improve outcomes in patients with acute HE. However, the effectiveness of BCAA administration during the acute phase remains unclear. This study aimed to evaluate the effect of intravenous BCAA (IV-BCAA) treatment on clinical outcomes in patients with acute HE by systematically reviewing and analyzing randomized controlled trials (RCTs).

**Methods:**

We conducted a comprehensive literature search of MEDLINE, the Cochrane Central Register of Controlled Trials, and Igaku Chuo Zasshi (ICHUSHI), a Japanese database for medical literature. We included RCTs involving adult patients with acute HE who received IV-BCAA or placebo during the acute phase after admission (< 7 days). Two reviewers independently screened the citations and extracted data. The primary “critical” outcomes were mortality from any cause and improvement in disturbance of consciousness. The secondary “important” outcome included the incidence of complications such as nausea and diarrhea. Risk ratios (RRs) were calculated using random effects models with inverse variance weighting.

**Results:**

Among the 2073 screened records, four met the criteria for quantitative analysis. The analysis included 219 patients: 109 received IV-BCAA, and 110 received placebo. Improvement in the disturbance of consciousness and mortality were not significantly different between the two groups (RR, 1.26; 95% confidence interval [CI], 0.96–1.66; RR, 0.90; 95% CI 0.70–1.16, respectively). Following IV-BCAA administration, the absolute differences of improvement in the disturbance of consciousness and mortality were 118 more per 1000 (95% CI 18 fewer–300 more) and 55 fewer per 1000 (95% CI 165 fewer–88 more), respectively. No significant differences were observed in the incidence of nausea or diarrhea between the two groups.

**Conclusions:**

Our meta-analysis demonstrates that all outcomes were not significantly different between IV-BCAA treatment and placebo for acute HE. Further RCTs are required to better understand IV-BCAA treatment potential in patients with HE.

**Supplementary Information:**

The online version contains supplementary material available at 10.1186/s40560-024-00771-x.

## Background

Hepatic encephalopathy (HE) is a complex neuropsychiatric syndrome resulting from acute liver failure. It is characterized by symptoms ranging from subtle cognitive impairments to severe alterations in consciousness, significantly impacting quality of life and increasing mortality rates. The pathogenesis of HE involves a combination of metabolic disturbances, including elevated serum ammonia levels, which disrupt neurotransmission and neuronal function [1].

The development of HE marks a significant transition in the natural history of cirrhosis. Following a diagnosis of HE, the median survival for persons with cirrhosis is substantially reduced to 2 years, 1 year if over 65 years old. HE occurs in as many as 40% of patients with cirrhosis [2]. Thus, cirrhosis was associated with 2.4% of global deaths [3].

Amino acids, particularly branched-chain amino acids (BCAAs), such as leucine, isoleucine, and valine, have been investigated for their potential therapeutic benefits in the management of HE. BCAAs are essential amino acids that are metabolized primarily in the muscles and influence brain function and neurochemistry [4]. The role of BCAAs in HE management stems from the hypothesis that BCAAs can provide neuroprotective effects and counteract some of the adverse effects of elevated ammonia levels [4]. An imbalance between BCAAs and aromatic amino acids (AAAs) is thought to contribute to the neurotoxic effects of HE. Thus, BCAAs may help restore balance by competing with AAAs for transport across the blood–brain barrier, potentially reducing the neurotoxic impact of AAAs.

Several studies have explored the efficacy of BCAA supplementation in improving HE symptoms, particularly cognitive function and overall clinical outcomes [5–9]. Compared with placebo or no intervention, oral BCAA supplementation in the chronic phase is more effective in treating overt HE [10]. However, evidence for this treatment in the acute resuscitative phase of HE with intravenous administration of BCAA (IV-BCAA) is lacking.

This systematic review and meta-analysis aimed to evaluate the existing evidence on the effectiveness of IV-BCAA administration for resuscitative acute HE treatment. By synthesizing data from multiple studies, we sought to determine whether IV-BCAA administration improves clinical outcomes in patients with acute HE, identifying potential benefits or limitations associated with this therapeutic approach.

## Methods

We organized a systematic review team in the Japan Resuscitation Council (JRC) Neuroresuscitation Task Force. The JRC 2025 Neuroresuscitation Task Force and the Guidelines Editorial Committee were established in 2024 and organized by the Japan Society of Neuroemergencies and Critical Care, the Japanese Society of Intensive Care Medicine, the Japan Society of Neurosurgical Emergency, and the Japanese Society of Neurological Therapeutics. The JRC Neuroresuscitation Task Force established six clinically relevant questions and performed a systematic review.

The systematic review was conducted in accordance with the Preferred Reporting Items for Systematic Reviews and Meta-Analyses standards [11]. This study was registered in the University Hospital Medical Information Network (UMIN) Clinical Trials Registry, the largest clinical trial registry in Japan (UMIN000054559).

Based on the discussion in the JRC Neuroresuscitation Task Force, the population intervention comparator outcome study design and timeframe to guide a systematic review search was set as follows:

- P (patients): All adults (≥ 18 years old) with HE.
- I (interventions): Initial administration of IV-BCAA during the in-hospital acute phase (< 7 days or in the acute hospitalized phase, not in outpatient clinics). The doses of BCAA used were not limited.
- C (comparisons, controls): Placebo or non-intervention.
- O (outcomes): Primary, “critical” outcome was mortality from any cause and improvement in consciousness. Secondary, “important” outcomes were nausea and diarrhea, which are common side effects.
- S (study design): Randomized controlled trials (RCTs).
- T (timeframe): All published literature up to June 4, 2024.

We identified RCTs investigating the effects of IV-BCAA in patients with acute HE by searching PubMed, the Cochrane Library, and Igaku Chuo Zasshi (ICHUSHI) web up till June 4, 2024. ICHUSHI web is the largest database of Japanese medical journals, containing approximately 10 million manuscripts from 6,000 journals.

We included studies that fulfilled the following criteria: (1) RCT, (2) full-text publications in English or Japanese, (3) included adult patients with acute, worsened HE, (4) included comparisons between IV-BCAA and placebo or non-intervention, and (5) initial administration of IV-BCAA during the acute phase (within 7 days after admission). The dose of administration and components of BCAA were not limited; however, the method of administration was limited to transvenous infusion because of the limitations of the acute resuscitation phase.

Two reviewers (TY and SY) independently extracted the data and assessed the methodological quality of eligible studies. They reached a consensus on literature selection, resolving disagreements through discussion. Data extracted from each study included the first author’s name, year of publication, number of study sites, number of patients, patient age, proportion of males, definition of HE, precipitating factors (infection, GI bleeding, and alcohol abuse), initial serum ammonia, administration of BCAA, dose of BCAA, and other treatments. Methodological quality was evaluated using the Cochrane risk-of-bias (RoB) assessment tool [12], which assesses the randomization process, derivations from intended interventions, missing outcome data, measurement of the outcome, and selection of the reported result. The two reviewers reached a consensus on RoB 2, and any discrepancy in judgment was resolved through discussion.

The grade of recommendation, assessment, development, and evaluation (GRADE) approach was also used to evaluate the certainty of the available evidence, such as inconsistency, indirectness, imprecision, and publication bias. We provided an evidence profile table using the GRADE pro GDT (GRADEpro GDT: GRADEpro Guideline Development Tool [Software]) and McMaster University, 2015 (developed by Evidence Prime Inc.). The table is available at gradepro.org. To apply the GRADE system, we received guidance from the Medical Information Network Distribution Service (MINDS), a Japanese center for GRADE education. The two reviewers also discussed the results of the RoB and reached a consensus on the final decision.

According to the GRADE approach, the JRC neuroresuscitation task force determines the importance of each outcome as critical, important, or not important [12, 13]. We defined critical outcomes as hospital mortality and improvements in disturbance of consciousness, and important outcomes as common side effects such as nausea and diarrhea.

We performed the meta-analysis using Review Manager (version 5.3, The Nordic Cochrane Centre, The Cochrane Collaboration, Copenhagen, Denmark). Comparative risk ratios (RRs) were reported with 95% confidence intervals (CIs). We selected a random-effects model. Statistical heterogeneity was determined by assessing values that were interpreted as follows: 0–40%, not important; 30–60%, moderate heterogeneity; 50–90%, substantial heterogeneity; and 75–100%, considerable heterogeneity.

## Results

### Literature search strategy

**Fig. 1:**
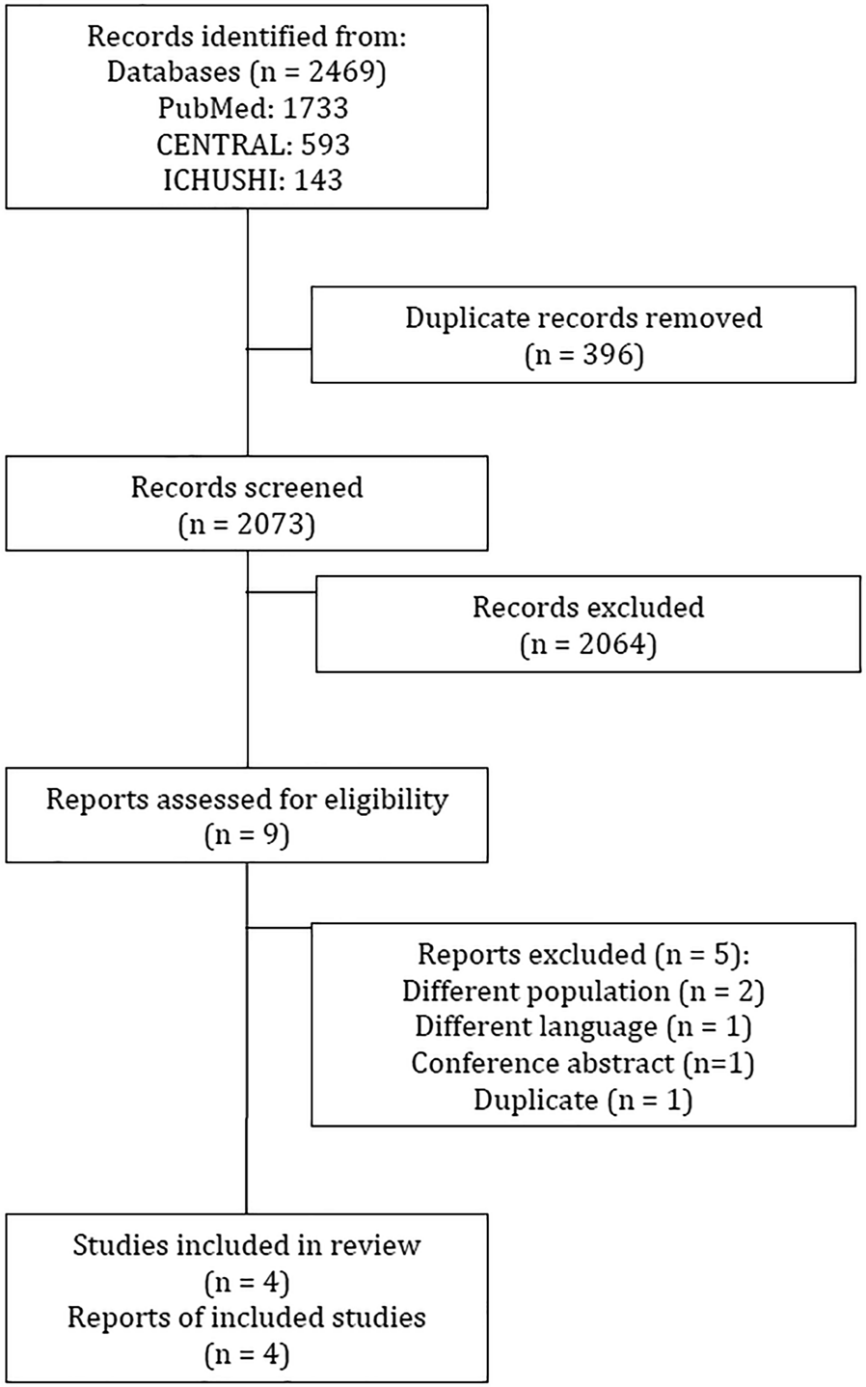
Flow chart of the search strategy and study selection

A total of 2469 studies were identified using the database search. After removing 396 duplicate studies, 2073 studies were eligible for the first screening. Based on the assessment of title and abstract, 2064 study records were excluded, and nine full-text articles were included for the second assessment as the full-text article assessment. After assessing the eligibility of the records, five reports were excluded for the following reasons; two studies were excluded because of different patient populations, one study was excluded because of different languages, one study was excluded because it was a conference abstract, and one study was excluded because of duplication. Thus, four RCTs were ultimately included in this meta-analysis (Fig. 1) [14–17]. The search formulae and results are listed in Additional file 1.

### Patient characteristics

The characteristics of studies included in this meta-analysis are summarized in Table 1. Overall, 219 patients selected from four RCTs were assigned to the IV-BCAA treatment group (*n* = 109) or placebo group (*n* = 110) for critical outcome analysis. Each study included 34–70 patients, with average age ranging from 45.3 to 58.9 years. The proportion of males was 56.0–88.6%. The largest RCT population was an open-label RCT published in 2023 [17]. Three studies were multicenter RCTs. In all studies, BCAA was administered intravenously. The most common precipitating factor was gastrointestinal bleeding (27.9%; n = 61).

**Table 1:** Characteristics of the included studies

Author, year	No. of sites	No. of patients (BCAA vs Control)	Mean Age (years)	Definition of HE	Male n, (%)	Precipitating factor, n (%)			Initial Mean Serum Ammonia	Intervention BCAA Type and dose	Methods of BCAA administration	Comparison/Control	Outcomes
						Infection	GI Bleeding	Alcohol Abuse					
Rossi-Fanelli et al. 1982 [14]	4	34 (17vs17)	58.9	Grade 3–4 (Adams and Foley classification)	21 (61.8)	12 (35.3)	7 (20.6)	1 (2.9)	172.4 (g/dL)	BS 692^a^ + 20% dextrose	60 ml/L for the first 24 h (1500 ml/day), and thereafter at 80 ml/hr (2500 ml/day) until 48 h after mental recovery	Lactulose via a nasogastric tube (30–40 g every 4 h) or via the rectal route (200–300 g/day). Dextrose in isocaloric amounts and the same rate of as in intervention group	Improvement in HE (Grade 0) during 48 h
Wahren et et al. 1983 [15]	5	50 (25vs25)	56	Grade II–IV (Denis classification)	28 (56.0)	12 (24.0)	20 (40.0)	2 (4.0)	2.4 times local laboratory’s upper normal limit	BCAA 20 g/L (70% leucine, 20% valine, and 10% isoleucine) in 5% glucose	2L of BCAAs (40 g)/ day. Infusions were given for 20 h/ day, and therapy was continued for 1 day after HE improved to Grade 0 or I, or for a maximum of 5 days	Glucose (5%) alone, 2 L/day	Improvement in HE (Grade 0–II) during 5 days 30-day mortality
Vilstrup 1990 [16]	3	65 (32vs33)	55.5	Grade II–IV (Fogarty classification)	47 (72.3)	15 (23.1)	20 (31.0)	3 (4.6)	N/A	1 g/kg/day of amino acid with 40% branched chain contents	Central venous infusion	Isocaloric 8% glucose	Improvement in HE (Grade 0–I) during 16 days 16-day mortality
Mehtani et al. 2023 [17]	1	70 (35vs35)	47	EASL-CLIF-defined ACLF with HESA grades > 2	62 (88.6)	19 (27.1)	14 (20.0)	28 (40.0)	134 (μmol/L)	BCAA 500 mL/day for 3 days + Lactulose 30–60 mL three times a day	Intravenous infusion	Lactulose alone 30–60 mL three times a day	Improvement in HE at day 28^b^ 28-day mortality

SD, Standard Deviation; HE, Hepatic Encephalopathy; GI, Bleeding, Gastrointestinal Bleeding; BCAA, Branched Chain Amino Acid; EASL-CLIF, European Association for the Study of the Liver-Chronic Liver Failure Consortium; ACLF, Acute-on-Chronic Liver Failure; HESA, hepatic encephalopathy scoring algorithm; hr, hours

^a^Composition of BCAA Mixture (BS692): L-Isoleucine 9 g/L, L-Leucine 11 g/L, L-Valine 8.4 g/L (Total BCAA 28.4 g). Dextrose 200 g/L, Total Nitrogen 4.54 g/L, Total calorie: 814.2 kcal/L

^b^Resolution of HE was defined as an improvement in HESA to grade 0 or 2 consecutive days when HESA grade remained at 1 after an initial improvement in at least 1 full grade

### Critical outcome: improvement in the disturbance of consciousness

**Fig. 2:**
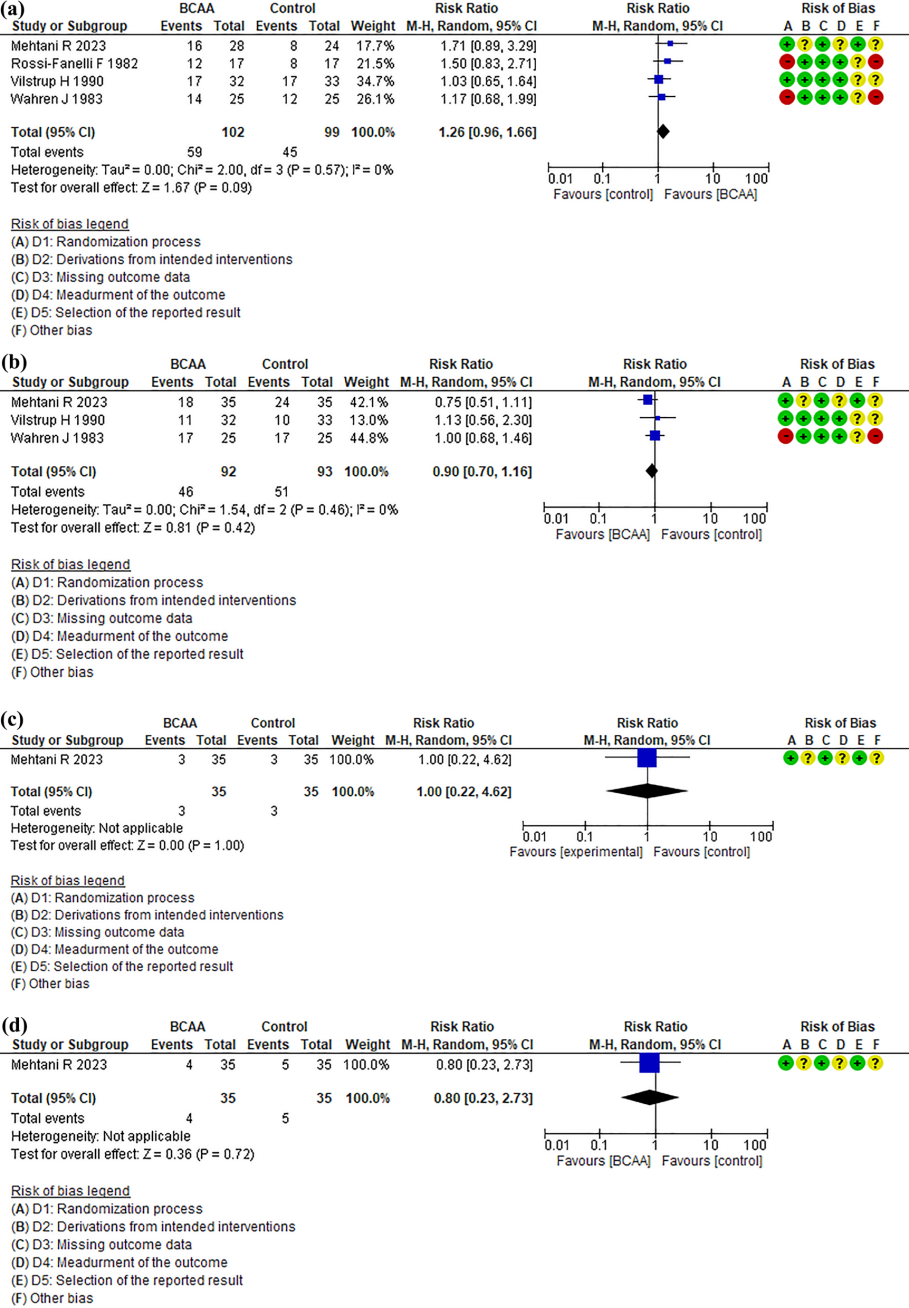
Forest plot of (**a**) Improvement in the disturbance of consciousness (**b**) All-cause mortality (**c**) Nausea, and (**d**) Diarrhea. The risk of bias summary is listed as follows: **A** D1 Randomization process; **B** D2 Derivations from intended interventions; **C** D3 Missing outcome data; **D** D4 Measurement of the outcome; **E** D5 Selection of the report result; **F** other bias. BCAA, branched-chain amino acid; CI, confidence interval

Improvement in the disturbance of consciousness was evaluated in all four RCTs (Fig. 2a). The evidence profiles are shown in Table 2. This set of four RCTs had less publication bias with a symmetric distribution in the funnel plot (Additional file 2a). The forest plot of one of the critical outcomes, improvement in the disturbance of consciousness, is shown in Fig. 2a. During the observation period, 59 patients (57.8%) in the BCAA-treated group and 45 patients (45.5%) in the placebo group recovered their mental status. Although this difference did not reach significance (RR, 1.26 [95% CI 0.96–1.66], P = 0.09; Fig. 2a), the absolute differences in improvement in the disturbance of consciousness were 118 more per 1000 (95% CI 18 fewer–300 more) as a result of BCAA administration.

**Table 2:** Evidence profiles

Certainty assessment							No of patients		Effect		Certainty	Importance
No. of studies	Study design	Risk of bias	Inconsistency	Indirectness	Imprecision	Other considerations	BCAA	placebo	Relative (95% CI)	Absolute (95% CI)		
Improvement of the disturbance of consciousness												
4	Randomized trials	Very serious^a^	Not serious	Not serious	Serious^b^	None	59/102 (57.8%)	45/99 (45.5%)	**RR 1.26** (0.96 to 1.66)	**118 more per 1000 **(from 18 fewer to 300 more)	⨁◯◯◯ Very low	CRITICAL
All-cause mortality												
3	Randomized trials	Very serious^c^	Not serious	Not serious	Serious^b^	None	46/92 (50.0%)	51/93 (54.8%)	**RR 0.90** (0.70 to 1.16)	**55 fewer per 1000** (from 165 fewer to 88 more)	⨁◯◯◯ Very low	CRITICAL
Nausea												
1	Randomized trials	Serious^c^	Not serious	Not serious	Serious^b^	None	3/35 (8.6%)	3/35 (8.6%)	**RR 1.00** (0.22 to 4.62)	**0 fewer per 1000** (from 67 fewer to 310 more)	⨁⨁◯◯ Low	IMPORTANT
Diarrhea												
1	Randomized trials	Serious^c^	Not serious	Not serious	Serious^b^	None	4/35 (11.4%)	5/35 (14.3%)	**RR 0.80 **(0.23 to 2.73)	**29 fewer per 1000** (from 110 fewer to 247 more)	⨁⨁◯◯ Low	IMPORTANT

Reasons of downgrade:

a. "There are two studies with a high risk of bias."

b. "There is a high possibility that the optimal amount of information is not met."

c. "There is one study with a high risk of bias, and its contribution rate is high."

CI: Confidence interval; RR: Risk ratio; RoB: Risk of Bias

### Critical outcome: all-cause mortality

Mortality from any cause was evaluated in three RCTs (Fig. 2b). The evidence profiles are presented in Table 2. This set of three RCTs had less publication bias with a symmetric distribution in the funnel plot (Additional file 2b). During the observation period, 46 patients (50.0%) died in the IV-BCAA-treated group, and 51 patients (54.8%) died in the placebo group. Although this difference did not reach significance (RR, 0.90 [95% CI 0.70–1.16], p = 0.42), the absolute differences in mortality were 55 fewer per 1000 (95% CI 165 fewer–88 more) as a result of IV-BCAA administration.

### Important outcome: adverse effects (nausea and diarrhea)

The incidence rates of the adverse effects of IV-BCAA treatment, nausea, and diarrhea were determined for each RCT [16] (Fig. 2c, d). With respect to nausea, three (8.6%) of the 35 patients who received BCAAs experienced nausea, and the same number (8.6%) of patients in the placebo group experienced nausea. No significant difference was observed in the incidence of nausea between the BCAA and placebo groups (RR, 1.00; 95% CI [0.22–4.62]; *P* = 1.00) (Fig. 2c).

Regarding the incidence of diarrhea, it occurred in four (11.4%) patients in the IV-BCAA group and five (14.3%) patients in the placebo group. No significant difference was observed in the incidence of nausea between the BCAA and placebo groups (RR, 0.80; 95% CI [0.23–2.73]; *P* = 0.72). (Fig. 2d). As a result of BCAA administration, the absolute differences in nausea and diarrhea were 0 fewer per 1000 (95% CI 67 fewer–310 more) and 29 fewer per 1000 (95% CI 110 fewer–427 more), respectively.

## Discussion

In this systematic review and meta-analysis, we aimed to clarify the efficacy of IV-BCAA administration compared to placebo in patients with acute HE. Several systematic reviews of BCAA treatment in HE patients have been published [8, 16–19]. However, in these meta-analyses, most RCTs did not mention acute HE, which requires urgent care and intravenous administration in the emergency room or intensive care unit. Therefore, this study is the first systematic review and meta-analysis of RCTs comparing IV-BCAA treatment and placebo in acute-phase HE care.

Our meta-analysis demonstrates that all outcomes were not significantly different between IV-BCAA treatment and placebo for acute HE. Therefore, the benefit of IV-BCAAs remains unclear. However, our meta-analysis indicated the possibility that IV-BCAA treatment might have potential benefits in this population because improvements in the disturbance of consciousness in the IV-BCAA group tended to be higher.

BCAAs, including leucine, isoleucine, and valine, play various roles in the body, which include supporting protein synthesis and energy production. With regard to the pathophysiology of HE, BCAAs play a crucial role in ammonia metabolism [21]. In patients with liver disease, the protein breakdown in muscles leads to an increase in ammonia levels. BCAAs can provide an alternative nitrogen source that is converted into glutamine in muscles, thereby reducing the level of toxic ammonia in the blood [20].

In HE, there is often an imbalance between neurotransmitters in the brain, particularly a decrease in excitatory neurotransmitters such as glutamate and an increase in inhibitory neurotransmitters such as gamma-aminobutyric acid [22]. BCAAs can also help restore this balance by serving as precursors of excitatory neurotransmitters, potentially improving cognitive function. Additionally, patients with liver disease are often malnourished, and BCAAs are important for maintaining muscle mass and overall nutritional status [23]. Several clinical studies have shown that oral BCAA supplementation can improve mental health and reduce the severity of HE symptoms via the above pathophysiological mechanisms [5, 24–26]. However, the benefits are often modest, and BCAAs are usually part of a broader treatment plan rather than a standalone solution [27]. BCAA supplementation is often used as an adjunct therapy in the management of chronic phase HE, particularly in patients who are unresponsive to standard treatments such as lactulose or rifaximin [28]. Thus, BCAA administration is usually transoral during the chronic phase of HE management [27]. Recent guidelines from the European Association for the Study of the Liver and American Association for the Study of Liver Diseases recommend routine oral BCAA treatment for patients with overt HE [29, 30]. However, the effect of IV-BCAA on episodic bouts of HE has not been proven [31].

Our findings support the safety of IV-BCAA in clinical practice because of the equal incidence of side effects in the IV-BCAA and placebo groups. However, we found only one RCT that mentioned side effects (nausea and diarrhea). Generally, the adverse effects of BCAA treatment are listed as hypoglycemia, nausea, and diarrhea [32].

Indeed, prophylactic glucose administration may be applied; however, the incidence of these adverse effects is still not well understood.

Moreover, we could not find sufficient evidence to evaluate the benefit of IV-BCAA compared with other interventions because improvements in the disturbance of consciousness and mortality were not significantly different between the two groups.

We also could not discuss the cost-effectiveness of the interventions for the management of HE because all four RCTs did not evaluate it. An economic study revealed that the total cost related to HE in the United States increased from USD 4676.7 million in 2005 to USD 7244.7 million in 2009 [33]. Therefore, it is necessary to assess the cost-effectiveness of IV-BCAA treatment for HE from the standpoint of medical economics, benefits, and harm.

This meta-analysis has some limitations. First, only four RCTs were included in our systematic review, with limited information on a small number of patients because of the inclusion criteria of patients who received IV-BCAAs in emergency care. Second, the severity of HE differed across the four RCTs; that is, the definition of the inclusion of patients with HE may have differed across the RCTs. Thus, the features of the targeted patients may have been heterogeneous in our meta-analysis. Finally, the underlying conditions that precipitated HE varied, as shown in Table 1. However, we could not perform a subgroup analysis based on these underlying conditions because the four RCTs did not provide data for each of these subgroups. Therefore, the balance between the risks and benefits of IV-BCAA might differ depending on the underlying conditions that precipitate HE. Well-designed RCTs are needed in the future to determine the indications for IV-BCAA in acute HE care and support our findings.

## Conclusions

Our meta-analysis demonstrates that all outcomes were not significantly different between IV-BCAA treatment and placebo for acute HE. However, improvements in the disturbance of consciousness in the IV-BCAA group tended to be higher. These findings highlight the need for further RCTs to better understand the potential benefits of IV-BCAA treatment in this population.

## Supplementary Information

**Additional file 1.** Search strategy and results.

**Additional file 2.** Funnel plot. (a) Critical outcome: Improvement in the disturbance of consciousness. (b) Critical outcome: All-cause mortali

**Additional file 3.**

## Data Availability

Not applicable.

## Abbreviations

HE: Hepatic encephalopathy
BCAA: Branched-chain amino acid
IV-BCAA: Intravenous BCAA treatment
RCTs: Randomized controlled trials
AAA: Aromatic amino acid
