# Supplementary figures and images for: Intravenous branched-chain amino acid administration for the acute treatment of hepatic encephalopathy: a systematic review and meta-analysis
Source: J Intensive Care. 2025 Jan 9;13:2. doi: 10.1186/s40560-024-00771-x (PMC11716518; doi:10.1186/s40560-024-00771-x)

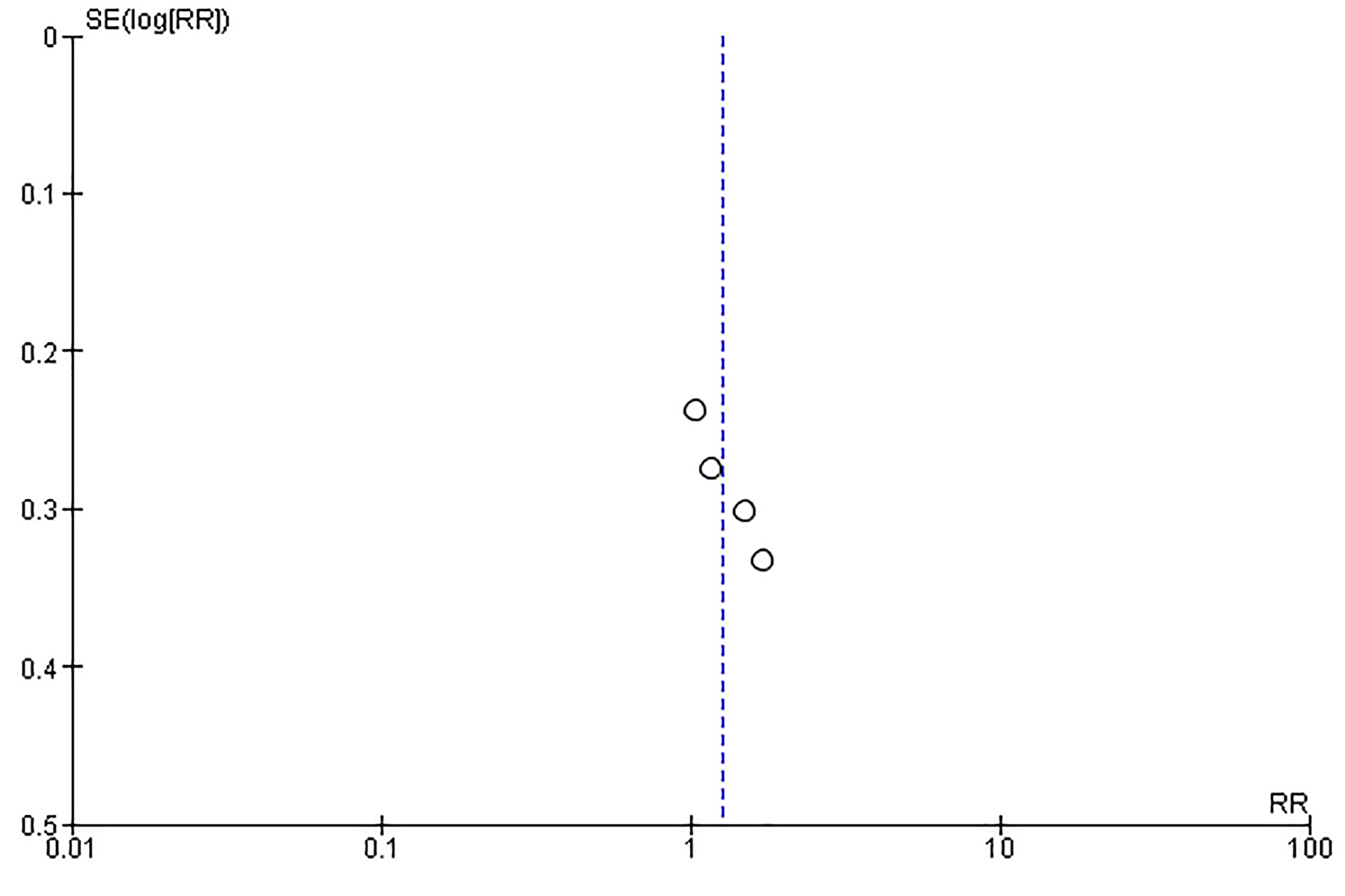

Supplement: Supplementary file 1 — Additional file 1. Search strategy and results. [file 40560_2024_771_MOESM1_ESM.tif]

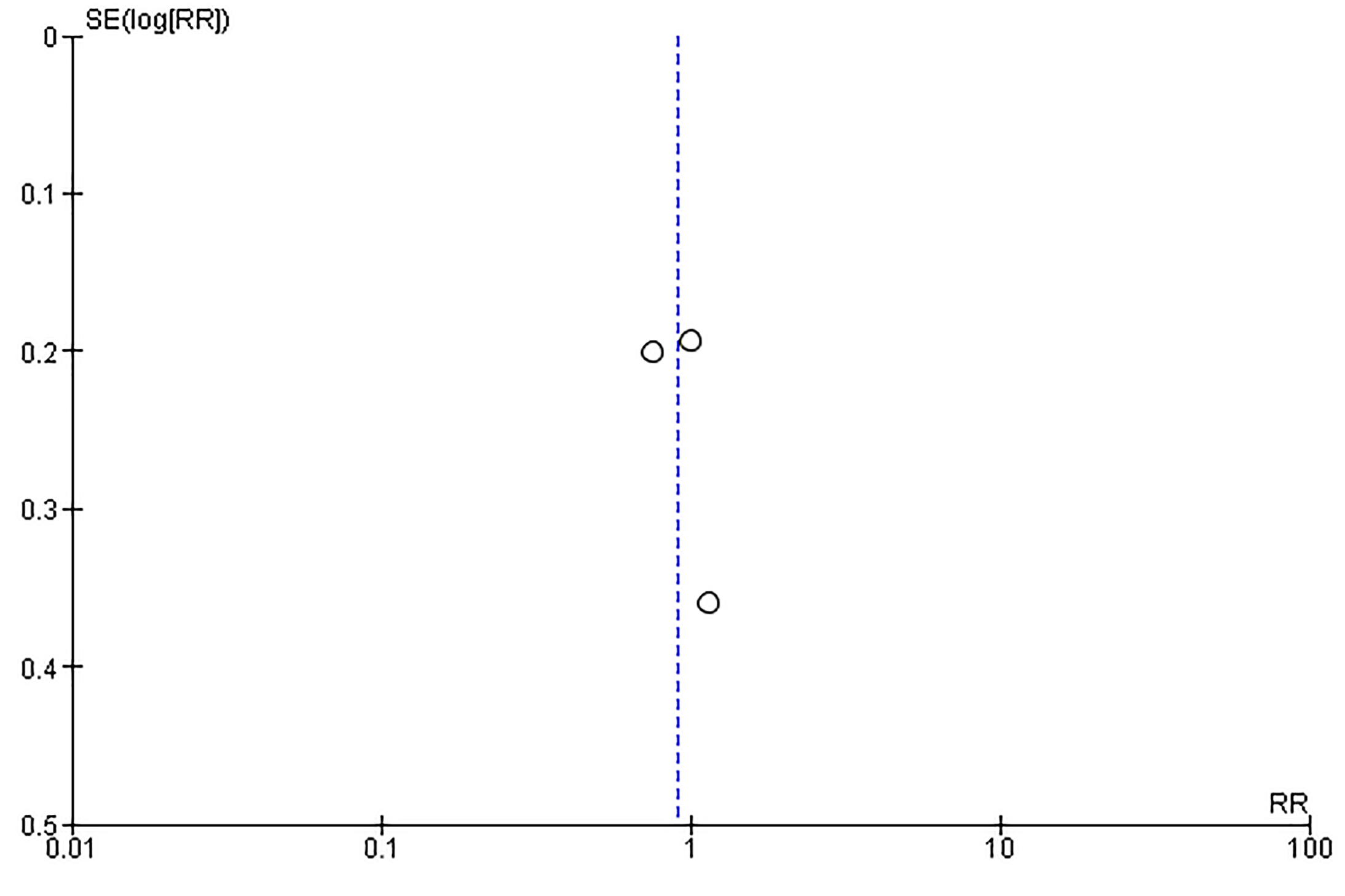

Supplement: Supplementary file 2 — Additional file 2. Funnel plot. (a) Critical outcome: Improvement in the disturbance of consciousness. (b) Critical outcome: All-cause mortali [file 40560_2024_771_MOESM2_ESM.tif]
