# Supplementary material for: Intravenous branched-chain amino acid administration for the acute treatment of hepatic encephalopathy: a systematic review and meta-analysis
Source: J Intensive Care. 2025 Jan 9;13:2. doi: 10.1186/s40560-024-00771-x (PMC11716518; doi:10.1186/s40560-024-00771-x)
Supplement: Supplementary file 3 — Additional file 3. [file 40560_2024_771_MOESM3_ESM.docx]

**Supplementary Table：Searching formulae and the results of search.**

PubMed search strategy（Date of search: 2024/6/4）

| #1 | "amino acids, branched chain"[MeSH Terms] OR "Branched-Chain"[Title/Abstract] | 64,425 |
| --- | --- | --- |
| #2 | "hepatic encephalopathy"[MeSH Terms] OR "liver diseases"[MeSH Terms] OR "fibrosis"[MeSH Terms] OR "encephalopath*"[Title/Abstract] OR "liver disease*"[Title/Abstract] OR "cirrho*"[Title/Abstract] | 863,177 |
| #3 | #1 and #2 | 3,646 |
| #4 | ((randomized controlled trial[pt] OR controlled clinical trial[pt] OR randomized[tiab]) OR placebo[tiab] OR drug therapy[sh] OR randomly[tiab] OR trial[tiab] OR groups[tiab] NOT (animals[mh] NOT humans[mh])) | 5,304,981 |
| #5 | #3 and #4 | 1,733 |

CENTRAL search strategy（Date of search: 2024/6/4）

| #1 | MeSH descriptor: [Amino Acids, Branched-Chain] explode all trees | 2905 |
| --- | --- | --- |
| #2 | (branched chain):ti,ab,kw | 1340 |
| #3 | #1 or #2 | 3777 |
| #4 | MeSH descriptor: [Hepatic Encephalopathy] explode all trees | 615 |
| #5 | MeSH descriptor: [Liver Diseases] explode all trees | 20947 |
| #6 | MeSH descriptor: [Fibrosis] explode all trees | 8306 |
| #7 | (encephalopath* or liver disease* or cirrho*):ti,ab,kw | 42876 |
| #8 | #4 or #5 or #6 or #7 | 56496 |
| #9 | #3 and #8 | 593 |

Igaku Chuo Zasshi (ICHUSHI) search strategy （Date of search: 2024/6/4）

| #1 | 肝硬変用アミノ酸製剤/TH or 肝不全用アミノ酸製剤/TH | 510 |
| --- | --- | --- |
| #2 | (("Branched-Chain Amino Acids"/TH or 分枝鎖アミノ酸/TA)) | 13094 |
| #3 | #1 or #2 | 13338 |
| #4 | 肝性脳症/TH | 5187 |
| #5 | 肝硬変/TH | 43063 |
| #6 | ((肝性脳症/TH or 肝性脳症/TA)) | 6280 |
| #7 | ランダム化比較試験/TH or 準ランダム化比較試験/TH or ランダム化/AL or 無作為化/AL or 比較試験/AL or 臨床試験/AL or プラセボ/AL or 対照/AL or コントロール/AL or 臨床研究/AL | 361537 |
| #8 | #4 or #5 or #6 | 47829 |
| #9 | #3 and #8 | 1778 |
| #10 | (#7 and #9) and (PT=会議録除く) | 143 |
